# CRP Stimulates GDF15 Expression in Endothelial Cells through p53

**DOI:** 10.1155/2018/8278039

**Published:** 2018-06-03

**Authors:** Yoonseo Kim, Nicole Noren Hooten, Michele K. Evans

**Affiliations:** Laboratory of Epidemiology and Population Science, National Institute on Aging, National Institutes of Health, Baltimore, MD, USA

## Abstract

Growth differentiation factor 15 (GDF15) is a multifunctional, secreted protein that is a direct target gene of p53. GDF15 is a prospective biomarker of cardiovascular disease (CVD). C-reactive protein (CRP), like GDF15, is implicated in inflammation and an independent biomarker of CVD. However, the molecular interactions between GDF15 and CRP remain unexplored. In women, we found a significant relationship between hsCRP and GDF15 serum and mRNA levels. *In vitro* treatment of cultured human aortic endothelial cells (HAECs) with purified CRP or transfection of a CRP plasmid into HAECs induced GDF15 expression. Dual-luciferase reporter assays confirmed that CRP significantly increased the levels of GDF15 promoter luciferase activity, indicating that CRP induces GDF15 transcription. Chromatin immunoprecipitation (ChIP) assays confirmed that p53 was recruited to both p53 binding sites 1 and 2 in the GDF15 promoter in response to CRP. We have uncovered a linkage between CRP and GDF15, a new clue that could be important in the pathogenesis of endothelial inflammation.

## 1. Introduction

In the United States, heart disease remains the leading cause of death with approximately 610,000 deaths yearly [1]. Although men bear a disproportionate burden of cardiovascular disease (CVD), it is often overlooked that almost as many women die from CVD. Currently, approximately one in four women in the United States dies from CVD [2].

Inflammation- and immune system-related pathways are known to be important underlying factors in many age-related chronic diseases with heightened attention to CVD [3]. The initiation as well as the promotion of atherogenesis is linked to inflammatory processes. Inflammatory biomarkers, particularly C-reactive protein (CRP) and growth differentiation factor 15 (GDF15), provide a unique opportunity to understand the role of inflammation in CVD especially in populations at a unique risk.

CRP, an independent predictor of cardiovascular events, is an acute phase reactant protein and a nonspecific marker of systemic inflammation. It is produced in the liver and is both a direct participant and a cofactor in the inflammatory process [4, 5]. Of special note, multiple epidemiologic studies have shown that women have higher levels of CRP than men perhaps indicative of higher levels of inflammation that may convey additional cardiovascular risk [6, 7]. CRP is involved directly or indirectly in many and varied molecular pathways including cellular adhesion in endothelial cells, monocyte activation, and the promotion and progression of atherosclerotic plaques [8–10]. In addition to inducing endothelial dysfunction, CRP triggers plaque rupture, augments hypoxia-induced apoptosis, activates the complement pathway, and is a mediator of atherothrombotic disease. As previously reported, we found that CRP was associated with the oxidative stress marker, 8-oxo-7,8-dihydro-2′deoxyguanosine (8-oxodG), and that CRP generates reactive oxygen species in women suggesting that oxidative stress and inflammation are important cofactors of the clinical risk imparted by CRP [11].

GDF15 initially was classified as a divergent member of the TGF-*β* cytokine family and is also referred to as macrophage inhibiting cytokine 1 (MIC-1) [12], placental transformation growth factor (PTGF-*β*) [13], prostate-derived factor (PDF) [14], placental bone morphogenetic protein (PLAB) [15], and NSAID-activated gene-1 (NAG-1) [16]. Recent data suggests that GDF15 is more closely related to the glial cell-derived neurotrophic factors (GDNFs) due to its binding to the GDNF receptor alpha-like (GFRAL) proteins [17–20]. GDF15 is a direct target gene of p53. It is highly expressed in the placenta during pregnancy but is expressed at low levels in most tissues at baseline. Numerous investigators have demonstrated that GDF15 is also expressed in response to cytokines and growth factors including interleukin-1*β* (IL-1*β*), TNF-*α*, angiotensin II, macrophage colony stimulating factor (M-CSF), and TGF-*β* [13, 21, 22].

While the exact function of GDF15 is not completely understood, higher levels of GDF15 are associated with increased cardiovascular risk. High serum levels of GDF15 have been associated with acute myocardial infarction and have been correlated with levels of other cardiovascular risk biomarkers including troponin-T, N-terminal probrain natriuretic peptide, and CRP possibly suggesting a link between GDF15 and inflammation [23]. GDF15 is associated with the development of several age-related diseases including heart failure, coronary artery disease, atrial fibrillation, diabetes mellitus, cancer, and cognitive impairment [24, 25].

GDF15 is not only a prospective biomarker of CVD but also an independent predictor of all-cause mortality [26–28]. While both CRP and GDF15 are established independent predictors of cardiovascular risk, only GDF15 is also an independent predictor of all-cause mortality. In fact, GDF15 may have a better prognostic value in healthy individuals because it is more tightly correlated to inflammatory processes like oxidative stress and ischemia [29]. There is some evidence that GDF15 is a better prognostic biomarker among women than men. Elevated GDF15 accurately predicts the likelihood of secondary cardiovascular events in women but not in men who have had a previous diagnosis of carotid atherosclerosis [30]. While GDF15 increases with age like CRP, another study showed that elevated levels of plasma GDF15 predict future cardiovascular events in elderly women but not as accurately in elderly men [31]. GDF15 had a higher baseline level among women who were participants in the Women's Health Study who eventually developed cardiovascular-related disease states including thrombosis, stroke, and myocardial infarction. In this study, GDF15 was modestly correlated with and additive to CRP in identifying those at risk for cardiovascular disease events [32].

Here, we investigated the molecular relationship between CRP and GDF15 in women. We report that GDF15 expression levels were increased by CRP via p53 binding to its promoter region in human aortic endothelial cells (HAECs). These data provide insight into the relationship between these two clinically relevant markers of inflammation that may help us develop approaches for modulating the negative effects of inflammation in CVD- and age-related diseases in women. Expanding our understanding of cardiovascular risk factors in women might enhance the use of widely applicable and easy to use biomarker tools to improve clinical care of women at risk.

## 2. Materials and Methods

### 2.1. Clinical Study Participants

We identified women who are participants in the National Institute on Aging's Healthy Aging in Neighborhoods of Diversity Across the Life Span Study (HANDLS) who had low- (<3 mg/L), mid- (>3–20 mg/L), or high- (>20 mg/L) high sensitivity C-reactive protein (hsCRP) levels. These groups (*n* = 39/group) were age and race matched. The mean age of this subcohort was 49.7 ± 8.1 years. HANDLS is an interdisciplinary, longitudinal, epidemiological study of age-related health disparities among a socioeconomically diverse cohort of African Americans and whites who resided at the baseline in the city of Baltimore [33]. This study has been approved from the National Institute of Environmental Health Sciences, NIH Institutional Review Board (IRB), and therefore, the principal investigators obtained written informed consent from all participants. Additional demographic and clinical information about this subcohort has been described in a previous publication [11].

### 2.2. GDF15 ELISA

Human GDF15 Quantikine ELISA Kit (R&D Systems) was used according to the manufacturer's directions. Briefly, serum (13 *μ*L) from women with low-, mid-, or high hsCRP (*n* = 39/group) was incubated in GDF15 antibody-coated microplates for 2 h, then washed and incubated with GDF15 antibody conjugated for 1 h. After washing, plates were incubated with color reagent (hydrogen peroxide-chromogen mix) for 30 min. The optical density of each well was determined using a microplate reader set to 570 nm. The concentrations were calculated according to the standards.

To measure GDF15 levels in conditioned media, we treated HAECs with 25 *μ*g/mL CRP for the indicated time points in growth media. Conditioned media was collected, centrifuged at 300 xg for 5 min and then syringe filtered through a 0.45 *μ*M filter. 50 *μ*L of conditioned media was used to measure GDF15 levels by ELISA.

### 2.3. GDF15 mRNA Level in Human PBMCs

We analyzed *GDF15* mRNA levels in a subcohort of women with low- and high hsCRP as described above. Gene expression was examined in those individuals who also had stored PBMCs. Nineteen white and 20 African American females with an average age of 49.7 ± 8.1 years were used for this analysis. RNA was isolated from PBMCs using TRIzol (Invitrogen) according to the manufacturer's instructions.

### 2.4. Cell Culture, Reagent, and Transfection

We cultured primary human aortic endothelial cells (HAEC) in EMB-2 supplemented with the EGM-2 SingleQuot Kit (Lonza; Walkersville, MD). The HeLa cells used in this study were grown in Dulbecco's modified Eagle's medium (DMEM, Invitrogen) and supplemented with 10% fetal bovine serum (FBS). We obtained highly purified human recombinant C-reactive protein without sodium azide and free of endotoxins from Trichem Resources Inc. We purchased control small interfering RNA (siRNA) and p53 siRNA from Santa Cruz Biotechnology and obtained pCMV6-control or pCMV6-CRP plasmids from Origene. We transfected siRNAs and plasmids using Lipofectamine-2000 (Invitrogen). We isolated RNA and protein from the cells 48 h after transfection.

### 2.5. RNA Isolation and RT-qPCR Analysis

TRIzol (Invitrogen) was used to isolate total RNA from cells according to the manufacturer's instructions. Reverse transcription (RT) was performed using random hexamers (Invitrogen) and SSII reverse transcriptase (Invitrogen); the abundance of transcripts was assessed by quantitative PCR (qPCR) analysis using the 2x SYBR Green Master Mix (Applied Biosystems). The following primers were used (forward and reverse, resp.): AGACATGTCGAGGAAGGCTTTT and TCGAGGACAGTTCCGTGTAGAA for *CRP*, CTACAATCCCATGGTGCTCA and TATGCAGTGGCAGTCTTTGG for *GDF15*, and GCTCCTCCTGTTCGACAGTCA and ACCTTCCCCATGGTGTCTGA for *GAPDH*. *GDF15* expression in PBMCs was normalized to the average of *HPRT* and *UBC* expression using gene-specific primers. The following primers were used (forward and reverse, resp.): AGATGGTCAAGTCGCAAGCT and GGGCATATCCTACAACAAACTTGTC for *HPRT* and ATTTGGGTCGCGGTTCTTG and TGCCTTGACATTCTCGATGGT for *UBC.*

### 2.6. Western Blot Analysis

In preparation for this analysis, we washed cells twice with 1x cold PBS; then cell extracts were lysed using 2x Laemmli sample buffer. Lysates were boiled and analyzed by SDS-PAGE. Subsequently, lysates were immunoblotted with anti-GDF15 (D2A3) (Cell Signaling), anti-CRP (C-term) (Millipore), and anti-p53 (DO-1) (Santa Cruz Biotechnology) antibodies and then reprobed with anti-actin (I-19) (Santa Cruz Biotechnology) antibodies as a loading control. GDF15 precursor levels are shown in immunoblots.

### 2.7. Cloning of GDF15 Promoter and Luciferase Reporter Assays

The luciferase constructs containing the GDF15 promoter were amplified from human genomic DNA (Promega). The following primers were used to generate each construct: pGL3-GDF15-a (−966/+70 clone): forward primer TCTAGAACTCTTGACGTCAGATGATC and reverse primer TGAGAGCCATTCACCGTCCTGAGTTC; pGL3-GDF15-b (−133/+70 clone): forward primer CACCCCCAGACCCCGCCCAGCTGTGGTCATTG and reverse primer TGAGAGCCATTCACCGTCCTGAGTTC; and pGL3-GDF15-c (−966/+41 clone): forward primer TCTAGAACTCTTGACGTCAGATGATC and reverse primer TGTGCAGGTTGCGGCTATGAGCTGGG. After PCR, each fragment was cloned into pGL3-Basic vector (Promega) digested with XhoI/HindIII restriction enzymes. HeLa cells were transfected with 1 *μ*g of the pGL3-Basic vectors containing different lengths of the GDF15 promoter together with 0.1 *μ*g of TK-Renilla reporter plasmid (Promega) as an internal control. After 24 h of transfection, cells were treated with or without 25 *μ*g/mL CRP for 18 h and RL and FL activities were measured using the Dual-Luciferase® Reporter Assay System (Promega) as per the manufacturer's instructions.

### 2.8. Chromatin Immunoprecipitation Assays (ChIP assays)

For ChIP experiments, HAEC cells (∼4 × 10^7^) were washed with PBS and treated with 1% formaldehyde (Sigma-Aldrich) in the medium for 10 min at room temperature, followed by an addition of glycine to a final concentration of 0.125 M for 5 min. Cells were then scraped into PBS and centrifuged at 10,000 ×g for 5 min at 4°C. Cells were resuspended in cell lysis buffer (0.5% SDS, 10 mM EDTA, 50 mM Tris–HCl, pH 8.1), sonicated, and centrifuged to obtain the supernatant containing chromatin. The supernatant was diluted five-fold in ChIP dilution buffer (0.01% SDS, 1.1% Triton X-100, 1.2 mM EDTA, 16.7 mM Tris HCl, pH 8.1, 167 mM NaCl) and incubated with anti-p53 antibodies (DO-1, Santa Cruz Biotechnology) or normal mouse IgG (Santa Cruz Biotechnology) at 4°C overnight followed by incubation with Protein A-coated Dynabeads® (Novex) at 4°C overnight. Immunoprecipitates were then washed consecutively for 10 min at 4°C with rotation in low-salt wash buffer (0.1% SDS, 1% Triton X-100, 2 mM EDTA, 20 mM Tris HCl, pH 8.1, 150 mM NaCl), then high-salt wash buffer (0.1% SDS, 1% Triton X-100, 2 mM EDTA, 20 mM Tris HCl, pH 8.1, 500 mM NaCl), then LiCl wash buffer (0.25 M LiCl, 1% NP-40, 1% deoxycholate, 1 mM EDTA, 10 mM Tris–HCl, pH 8.1), and lastly in 1x TE buffer (10 mM Tris–HCl, 1 mM EDTA, pH 8.1). The complexes were eluted by adding elution buffer (10 mM Tris–HCl, pH 8, 300 mM NaCl, 55 mM EDTA, 0.5% SDS). The purified ChIP products were subjected to qPCR analysis using the 2x SYBR Green Master Mix (Applied Biosystems). DNA mixtures purified from aliquots of each chromatin sample were also subjected to qPCR analysis as input samples, and the results were presented as the ChIP-qPCR measurements normalized to their respective input levels. The following primers were used (forward and reverse, resp.): CATCTGGTCAGTCCCAGCTCAGA G and GCAACTCTCGGAATCTGGAG TCTTCG for p53 site1, AGGTATTGCCATCTTGCCCAGACTTG and GCTCACCTTGAAGCCATCCTCACAG for p53 site2, and AGGCTGGAATGGTGTCCTC and TAGGGGGAGG ATCTTTAGGTG for p53 nonbinding (NB) site [34].

## 3. Results

### 3.1. Comparative Expression of hsCRP and GDF15

**Figure 1:**
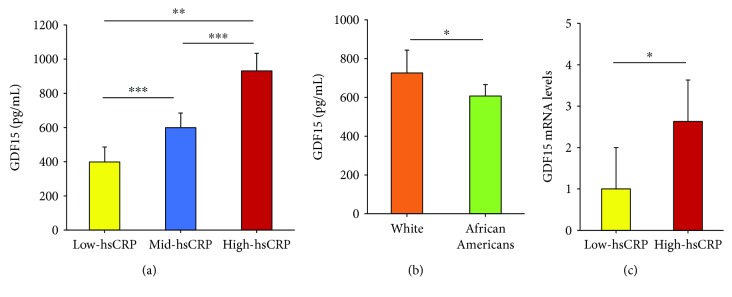
Women with hsCRP have high levels of GDF15. (a, b) Serum GDF15 levels were quantified by ELISA from HANDLS participants with either low- (<3 mg/L), mid- (>3–20 mg/L), or high hsCRP (>20 mg/L) levels (*n* = 39/group). The ELISA assay was performed according to manufacturer's instructions and was repeated in 2 independent experiments. (c) RNA was isolated from PBMCs from HANDLS participants with either low- (<3 mg/L) or high hsCRP (>20 mg/L) levels (*n* = 15/group). *GDF15* mRNA was quantified by RT-qPCR and normalized to *HPRT1* and *UBC* levels. The histograms represent the mean + SEM from three independent experiments. ^∗^*p* < 0.05, ^∗∗^*p* < 0.01, and ^∗∗∗^*p* < 0.001 by Student's *t*-test.

To examine the relationship between hsCRP and GDF15, we studied a cohort of diverse women from the HANDLS study with low- (<3 mg/L), mid- (>3–20 mg/L), or high hsCRP levels (>20 mg/L). Each group contained 39 women, and the groups were age (mean age, 49.7 ± 8.1 years) and race (19 whites, 20 African Americans) matched [11]. We measured serum GDF15 levels by ELISA and found a step-wise significant increase in GDF15 levels with increasing levels of hsCRP (Figure 1(a)). Since this cohort contains whites and African Americans, we also assessed whether there were differences in GDF15 levels by race. White women had higher levels of GDF15 than African American women (Figure 1(b)). To test whether *GDF15* mRNA levels were also higher in individuals with high hsCRP, we obtained peripheral blood mononuclear cells (PBMCs) from HANDLS participants with either low (<3 mg/L) or high (>20 mg/L) circulating protein levels of hsCRP [35]. *GDF15* mRNA levels were higher in women with high hsCRP (Figure 1(c)). These data suggest a positive correlation between the inflammatory markers hsCRP and GDF15 in women.

### 3.2. CRP Upregulates GDF15 in Endothelial Cells

**Figure 2:**
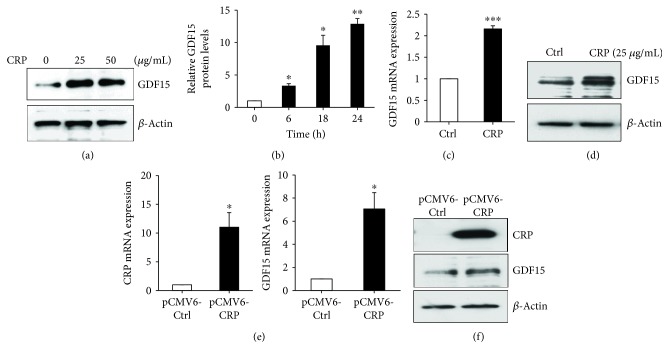
CRP upregulates GDF15 expression. (a) 18 h after CRP treatment with the indicated doses, GDF15 expression in HAECs was analyzed by immunoblotting with anti-GDF15 antibodies. *β*-Actin was used as a loading control. (b) After CRP treatment for the indicated time points, conditioned media was collected and GDF15 secreted levels were analyzed by ELISA. GDF15 levels were normalized to the 0 h time point for each experiment. The mean of three independent experiments is shown. (c and d) 18 h after CRP (25 *μ*g/mL) treatment, HAECs were lysed and levels of GDF15 mRNA or protein were quantified by RT-qPCR analysis (c) and western blot analysis (d). (e) HAECs were transfected with pCMV6-control or pCMV6-CRP plasmid for 48 h. Total RNA was isolated, and mRNA levels were quantified using RT-qPCR and normalized to *GAPDH*. (f) Total cell lysates from the indicated transfected HAECs were analyzed by Western blotting. *β*-Actin was used as a loading control. The histograms represent the mean + SEM from three independent experiments. ^∗^*p* < 0.05, ^∗∗^*p* < 0.01, and ^∗∗∗^*p* < 0.001 by Student's *t*-test.

Under normal physiological conditions, GDF15 is weakly expressed in most tissues. In response to multiple cellular stressors, such as acute injury, inflammation, and cancer, GDF15 expression can be dramatically induced [25, 36]. Given the positive correlation between hsCRP and GDF15 levels we found in women, we tested whether GDF15 expression can be induced in response to CRP exposure. We treated human aortic endothelial cells (HAECs) with a prolonged exposure (18 h) to highly purified recombinant CRP and subsequently analyzed GDF15 mRNA and protein expression *in vitro*. Treatment with CRP increased GDF15 expression in HAECs in a dose-dependent manner (Figure 2(a)). We also examined the levels of GDF15 secreted into the media by ELISA. CRP induced a time-dependent increase in GDF15-secreted levels (Figure 2(b)). To address whether CRP affects GDF15 at the transcriptional or posttranscriptional level, we analyzed *GDF1*5 mRNA levels by RT-qPCR. Both GDF15 protein and mRNA levels were upregulated by CRP treatment (Figures 2(c) and 2(d)). To confirm our results obtained by treatment with purified CRP, we overexpressed a plasmid containing CRP in HAEC cells. Consistent with our results with purified CRP, CRP overexpression increased *GDF15* mRNA and protein levels (Figures 2(e) and 2(f)). These results suggest that CRP can induce GDF15 expression in HAEC cells and are consistent with our *in vivo* data demonstrating a positive correlation between GDF15 expression and CRP expression in women.

### 3.3. CRP Promotes the Transcription of GDF15 via p53 Binding Sites in Its Promoter Region

**Figure 3:**
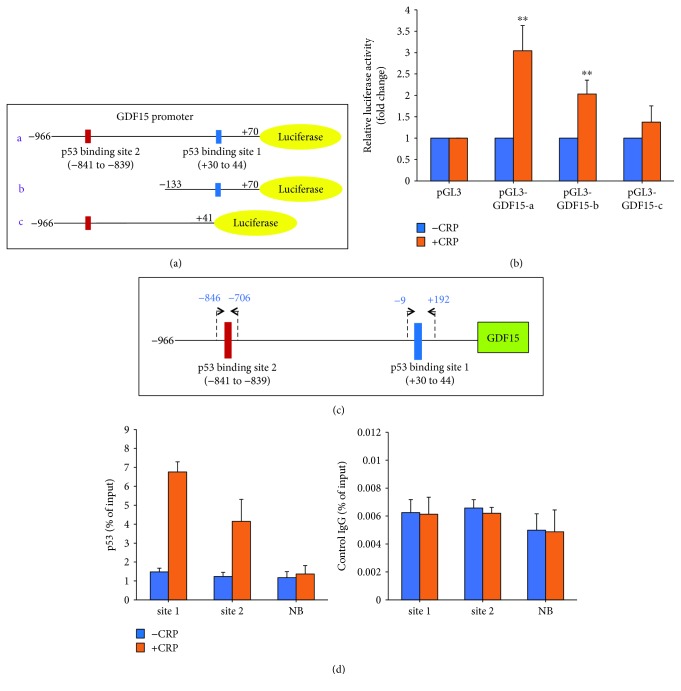
CRP promotes *GDF15* transcription. (a) Schematic of GDF15 promotor dual-luciferase constructs. Two p53 binding sites are indicated. (b) The indicated plasmids (1 *μ*g) were cotransfected with 0.1 *μ*g of TK-Renilla reporter plasmid in HeLa cells, and 24 h later, the cells were treated with CRP. After 18 h, the promoter activities were measured by luciferase activity. Transfection efficiency for luciferase activity was normalized to the Renilla luciferase activity. The results show the mean + SEM of three independent transfections. ^∗∗^*p* < 0.01 by Student's *t*-test. (c) Schematic of p53 binding sites and primers used for ChIP assays in the GDF15 promoter. (d) ChIP assays were performed on HAECs transfected for 24 h and treated with or without CRP for 18 h. DNA immunoprecipitated by antibodies to p53 or immunoglobulin G IgG (control) was amplified by qPCR. Each qPCR reaction was performed in triplicate, and the histogram represents the average of three independent ChIP assays + SEM.

Given that CRP affects both the mRNA and protein level of GDF15, we hypothesized that CRP may regulate GDF15 transcription. We thought this was possible because GDF15 contains two p53 binding sites in its promoter region and is a direct target gene of p53 [13, 37]. To investigate whether CRP induces GDF15 expression through its promoter, we performed dual-luciferase reporter assays. We cloned the *GDF15* promoter (−966/+70) by PCR into the pGL3-Basic vector (Figure 3(a)). The full-length construct contains two p53 binding sites (1 and 2) [38]. We also generated constructs containing the individual p53 binding site to analyze which site may be important for GDF15 regulation by CRP. pGL3-GDF15-b (−133/+70) construct contains p53 binding site 1, and pGL3-GDF15-c (−966/+41) construct contains p53 binding site 2 (Figure 3(a)). We transfected the Renilla reporter plasmid as a transfection efficiency control. After 18 h treatment with CRP, we analyzed luciferase reporter activity and the ratio of RL/FL was calculated for each transfected reporter plasmid. CRP significantly increased the levels of full-length GDF15 promoter luciferase activity, indicating that CRP induces GDF15 transcription. Furthermore, pGL3-GDF15 b and c luciferase activities were both increased (Figure 3(b)), indicating that p53 binding sites 1 and 2 are both critical sites for CRP-induced GDF15 expression.

To further investigate how CRP increases the expression of GDF15, we examined the interaction of p53 protein with endogenous *GDF15* gene promoter using chromatin immunoprecipitation (ChIP) assays. Our ChIP assays confirmed that p53 is recruited to both binding sites 1 and 2 in the *GDF15* promoter in response to CRP. As a negative control, we included a site where p53 is not predicted to bind. p53 was not recruited to the nonbinding site in our ChIP assays. These data suggest that CRP regulates GDF15 transcription via p53 binding to its promoter region.

### 3.4. Induction of GDF15 Expression Is p53-Dependent

**Figure 4:**
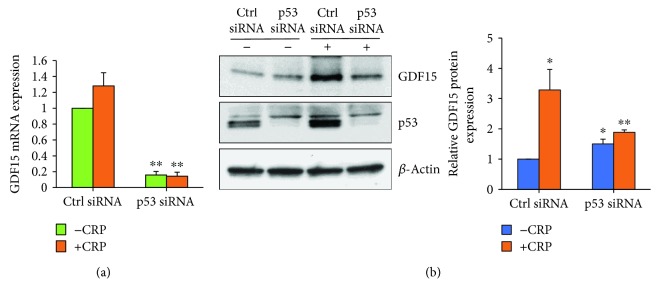
p53 knockdown inhibits CRP-induced GDF15 expression. HAECs were transfected with either Ctrl siRNA or p53 siRNA for 24 h and treated with or without CRP for 18 h. GDF15 mRNA levels were examined by RT-qPCR (a), and protein levels were analyzed by Western blot analysis (b). The histogram represents the mean + SEM from three independent experiments. ^∗^*p* < 0.05 and ^∗∗^*p* < 0.01 by Student's *t*-test.

To address whether p53 is required for CRP-mediated upregulation of GDF15, we silenced p53 using small interfering RNA (siRNA) and treated cells with CRP (Figures 4(a) and 4(b)). As shown in Figure 4(b), CRP treatment induces p53 accumulation in HAEC cells. However, p53 silencing significantly decreased GDF15 mRNA and protein levels in the presence of CRP. Taken together, these results suggest that CRP-mediated induction of GDF15 expression is dependent on p53.

## 4. Discussion

Although previous epidemiologic reports have suggested a link between GDF15 and CRP, existing data is scarce about the molecular pathways connecting these two biomarkers of inflammation. Given that both cardioprotective and proinflammatory roles have been described for GDF15, we wanted to further understand the relationship of this protein to CRP. Our data both *in vitro* and *in vivo* indicate a positive relationship between these two CVD markers.

Here, we found a significant positive correlation between hsCRP and GDF15 levels in a white and African American cohort of middle-aged women. Circulating GDF15 protein levels and mRNA levels in PBMCs were higher in women who also had hsCRP levels. To investigate this further, we used *in vitro* cell culture models and found that CRP induced GDF15 mRNA and protein levels in HAECs in a p53-dependent manner.

Previous epidemiological studies have indicated a potential relationship between GDF15 and CRP [25]. Investigators in the Rancho Bernardo study found a correlation between GDF15 and hsCRP values; higher GDF15 levels were associated with hsCRP levels [39]. Other data from cohort studies produced similar findings including a study of CVD events in women [32], in patients with coronary heart disease [40] and in atherosclerosis patients on hemodialysis [41]. While both biomarkers predict CVD events, there are distinct differences between CRP and GDF15. GDF15 is predictive not only of cardiovascular disease risk but also of longevity, healthy behaviors, incident cancer, cancer mortality, and biologic age [42]. So, even though our data shows a molecular link, this is likely only part of the story since the body of epidemiologic data suggests that CRP and GDF15 may be biomarkers that represent overlapping as well as nonoverlapping conditions.

In the context of understanding their interaction in cardiovascular disease risk, there are several factors to consider. CRP has a direct effect on promoting atherosclerotic processes via both endothelial and smooth muscle cell activation [43]. Furthermore, we have reported that CRP may contribute to cardiovascular disease by also increasing oxidative stress and DNA damage. Previously, we investigated the role of brain-derived neurotrophic factor (BDNF) in relation to CRP and found that BDNF has a cardioprotective role by inhibiting CRP expression and CRP-induced DNA damage [44]. Our previous work has shown that CRP generates intercellular ROS and induces at least one specific form of oxidative DNA damage, 8-oxodG, in vascular endothelial cells [11]. Early studies on GDF15 focused on the role of GDF15 in macrophage activation, growth inhibition, and apoptosis in tumor cells [12, 13, 37]. A more recent work has delineated a role for GDF15 in centrally regulating body weight, food intake, appetite, metabolism, and energy expenditure in both rodents and humans through coreceptors GFRAL (glial-derived neurotrophic family receptor *α* like) and the tyrosine kinase protein receptor RET, especially in response to tissue stress and injury [17, 18]. This new work attributing modulation of metabolism to GDF15 may provide new insights into its dual predictive values in cardiovascular disease and all-cause mortality. From the perspective of cardiovascular disease, previous work demonstrates that the peripheral expression of GDF15 is tightly regulated and induced in cardiovascular cell types under specific stress stimuli. GDF15 expression is regulated differently for diverse types of stress (endogenous, environmental) and the cell type and occurs through several transcriptional pathways including but not limited to HIF-1*α*, ATF-3, and KLF-4 [45–47]. For example, antiangiogenic stress can induce GDF15 in endothelial cells exposed to compounds that generate antiangiogenic stress, including nutlin-3 and N-(4-hydroxyphenyl) retinamide [48, 49]. GDF-15 can also be induced in vascular smooth muscle cells by stimulation with triglyceride-rich lipoproteins [50]. Two groups of investigators have demonstrated that adipocytes can synthesize and secrete GDF15 when exposed to oxidative stress. They may release paracrine factors that stimulate the expression of GDF15 in adjacent cells and tissues [51, 52]. GDF15 is also upregulated in mice after coronary arterial ligation [21]. Thus, GDF15 is produced by multiple cardiovascular cell types under stressful conditions so its correlation with the well-validated cardiovascular disease risk biomarker CRP is not surprising. Oxidative stress due to tissue injury or inflammation is an important inducer of GDF15 and CRP.

The other important link between CRP and GDF15 is through p53. GDF15 is a direct transcriptional target of p53. GDF15 is induced and secreted upon activation of p53 similar to p21. While being the most notable for its role in cancer, p53 also plays a fundamental role in aging, life span, the response to stress, and overall fitness. It is pivotal in many age-related diseases including cardiovascular disease where p53 induction has been associated with plaque instability [32]. In addition, p53 has been shown to be a key regulator of the cardiac transcriptome [53]. A study examining the induction of GDF15 induction in endothelial cells exposed to high glucose levels found that p53 played an important role [54], since there was evidence in the literature that CRP increased p53 protein levels and induced p53-mediated cell cycle arrest in H9c2 cardiac myocytes and monocytes [55, 56]; we focused on whether CRP induces GDF15 through p53. The GDF15 promoter contains two p53 binding sites, and here, using luciferase reporter assays and ChIP assays, we found that CRP induces GDF15 expression through the regulation of p53 binding sites in the GDF15 promoter. In addition, we also showed that knockdown of p53 inhibited CRP-induced GDF15 expression. So not only does CRP induce p53; this induction of p53 is a key factor in GDF15 induction and likely undergirds the predictive role that GDF15 may have in cancer and all-cause mortality.

## 5. Conclusions

Our data provide a molecular link between two biomarkers, GDF15 and CRP. These findings may facilitate improved understanding of the pivotal inflammatory pathways important in cardiovascular disease. These data provide insight into the relationship between these two clinically relevant markers of inflammation that may help us develop approaches for modulating the negative effects of inflammation in CVD and perhaps even other age-related diseases.
